# The Effects of Supraharmonic Distortion in MV and LV AC Grids

**DOI:** 10.3390/s24082465

**Published:** 2024-04-11

**Authors:** Andrea Mariscotti, Alessandro Mingotti

**Affiliations:** 1 Department of Electrical, Electronic and Telecommunications Engineering, and Naval Architecture (DITEN), University of Genova, 16145 Genova, Italy; 2 Department of Electrical, Electronic, and Information Engineering (DEI), University of Bologna, 40136 Bologna, Italy; alessandro.mingotti2@unibo.it

**Keywords:** accuracy, aging, distortion, disturbance, power losses, power quality, supraharmonics

## Abstract

Since the integration of electronic devices and intelligent electronic devices into the power grid, power quality (PQ) has consistently remained a significant concern for system operators and experts. Maintaining high standards of power quality is crucial to preventing malfunctions and faults in electric assets and connected loads. Recently, PQ studies have shifted their focus to a specific frequency range, previously not considered problematic—the supraharmonic 2 kHz to 150 kHz range. This range is not populated by easily recognizable harmonic components of the 50 Hz to 60 Hz mains fundamental, but by a combination of intentional emissions, switching non-linearities and byproducts, and various types of resonances. This paper aims to provide a detailed analysis of the impact of supraharmonics (SHs) on power network operation and assets, focusing on the most relevant documented negative effects, namely power loss and the heating of grid elements, aging of dielectric materials, failure of medium voltage (MV) cable terminations, and interference with equipment and power line communication (PLC) technology in particular. Under some shareable assumptions, limits are derived and compared to existing ones for harmonic phenomena, providing a clear identification of the primary issues associated with supraharmonics and suggestions for the standardization process. Strictly related is the problem of grid monitoring and assessment of SH distortion, discussing the suitability of normative requirements for instrument transformers (ITs) with a specific focus on their accuracy.

## 1. Introduction

Supraharmonic (SH) distortion consists of a set of phenomena of conducted emissions from distorting loads interacting with the feeding network and its elements: other loads, cables, transformers, etc.Such distortion is conventionally located in the 2 kHz to 150 kHz frequency interval as an extension of the harmonic frequency interval that is limited to 2 kHz for 50 Hz systems.

Supraharmonics are not only intended to be “faster” harmonics, but their propagation and aggregation characteristics are different, as well as their time behavior (e.g., duration, intermittency, etc.).

This has motivated an intense research effort in the last decade to provide a characterization similar to the one traditionally developed for harmonics. Depending on the characteristics of the respective sources, such emissions may have varied and peculiar time-frequency and impedance behavior. Paired with the relative difficulty of carrying out comprehensive tests, authors have concentrated mostly on relevant types of sources, such as wind parks [1,2], photovoltaic (PV) parks [3,4], electric vehicle (EV) chargers [5,6], and smart lighting (e.g., LED or fluorescent lamps) [7,8], including the various cases of lighting as victims and sources of secondary emissions [9,10].

In particular, in [1,11] the aggregation of multiple identical sources is studied in a wind park and PV park, respectively, accounting for the attenuation and phase rotation of connecting electrical elements and slight differences in the respective switching frequencies of the interfacing converters. The studies are, however, limited to the harmonic interval and the first portion of the SH interval. The results confirm that harmonics may be synchronized to the mains frequency, but high-frequency components have a nearly random phase distribution, resulting thus in partial cancellation and the known rms summation of individual emissions.

Individual emissions may vary with frequency depending on the relationship of the internal converter impedance and grid impedance, also affected by network resonances, as studied in [3,4] for PV inverters and [5,12] for EV chargers on a DC grid. In [4], it is underlined how much input capacitors of the nearby loads can attract SH emissions that do not consequently reach the point of common coupling with the grid and do not propagate much. This, of course, reduces disturbance to the distant connected equipment, but increases the stress on equipment capacitors, causing aging and premature failures.

Studied scenarios have considered predominantly low-voltage (LV) grids for a matter of convenience during test campaigns in terms of safety, availability of accessible sites, and variety and number of connected sources. Wind parks, due to the large installed power, are always found to be connected at the medium voltage (MV) level.

Apart from the understanding and characterization of the behavior of SH sources, the most pressing objectives are the definition of suitable limits and the identification at the same time of appropriate quantification methods, as SHs have negative effects on grids and grid components.

The adoption of more or less strict limits of SH distortion depends on the severity of the negative effects that need first to be documented and quantitatively assessed. Such negative effects can be seen as an extension of those attributed to harmonic distortion; they range from stress to dielectrics and insulating materials within cables, transformers, and capacitors, including the aging of filter components, to network resonance scenarios, and to various cases of interference, in particular to power line communication (PLC) devices. Another area of concern is human exposure to the electromagnetic field, including interference with implantable devices, as caused by high-frequency components such as SHs [13]. The topic is critical, being related to human health and safety; however, the magnetic field limit values identified by various standards and publications are always above a fraction of A/m for non-critical effects and several A/m for more serious consequences. This translates into hundreds of mA for common-mode current components and several A for differential-mode current components, de facto much larger than what is observed in practice and discussed in the following.

In some cases, published studies focusing on the harmonic interval may be extended by similarity to the lower portion of the SH interval, such as models for skin effect and proximity effect in conductors [14], as well as to some extent dielectric losses [15], as discussed in Section 2.

Phenomena of stress in dielectrics occurring at large electric field intensity within the material [16,17] are of course specific to high nominal voltage values and thus limited to MV grids. Accelerated partial discharge events are specific to AC grids and may occur in principle at both LV and MV levels, although again they are favored by high electric field values. The final consequence of such phenomena is the aging of dielectrics [15,17,18], with some catastrophic phenomena; for example, with MV cable terminations with a mix of dielectric stress and local heating as root causes [19,20].

Modern grids see a variety of loads featuring controlled static power converters interfaced through filters, leading to quite complex scenarios of network resonances [12,21,22]. Resonance may occur network-wide, e.g., all along one or more branches of a distribution grid, up to the point of common coupling (PCC) or intake, or locally to a subset of the grid, where some loads (including renewable energy sources) exchange reactive power through, e.g., filter units.

The phenomena exemplified above, apart from being concerned by the wide frequency span of the SH interval, also have a complex time-frequency pattern. Time duration and intermittency of SH emissions may be widely variable and depend not only on the operating conditions of the SH source, but also on the interaction with other connected devices nearby [23,24] and on the quality of the feeding voltage [25,26].

The perspective of this work is on the negative effects rather than on the sources and the aggregation or propagation of SH emissions. The objective is to provide an overview of the documented interaction with grid elements and connected devices, where stress, aging, and disturbance take place (Section 2). To this aim, this work has wider coverage than the two other publications aiming at defining limits for SH distortion: ref. [27] focused on audible noise, interference with residual current devices and flicker, possibly taking inspiration from [28]; ref. [29] proposed a separation of the operating frequency intervals for PLCs and power converters operating in DC grids, increasing the immunity test levels focusing then on electric arcs and signal-to-noise ratio requirements for correct detection.

Limits may then be derived under suitable assumptions (e.g., number of concomitant sources, grid impedance, etc.), discussing the relevance of the discussed phenomena (Section 4).

To show the representativeness of the discussed literature works and standards, a short bibliographic analysis also appears in Section 3.

## 2. Negative Effects of Supraharmonics

In the introduction, the negative (or detrimental) effects of supraharmonics were briefly reviewed and may be synthesized as follows:

- Power losses in conductors, due to frequency-dependent phenomena, such as skin effects and proximity effects;
- Aging of insulating materials (mainly in cables and transformers) due to local losses and self heating;
- Aging of capacitors with a combination of effects of dielectric stress (similarly to what occurs to insulating materials) and increased wiring losses (especially for large power capacitors);
- Specific damage to MV cable terminations caused by local heating and electric field gradient;
- Triggering of network resonances (impacting on the resulting voltage or current distortion of primary emissions), including local resonance phenomena between connected loads and apparatuses (favored, e.g., by the extensive use of interface and EMC filters) associated with the so-called “secondary emissions”;
- Interference with equipment, in particular, connected at the LV level, consisting of, e.g., domestic appliances, information technology (IT), lighting, energy meters, residual current devices, etc.;
- Flicker phenomena on LED and fluorescent lamps as a slightly different form of interference, causing visual disturbance to people;
- Specific interference with power line communication (PLC) circuits, more and more commonly used at LV but also at MV levels; for example, to exchange information on energy metering and for control purposes.

**Figure 1:**
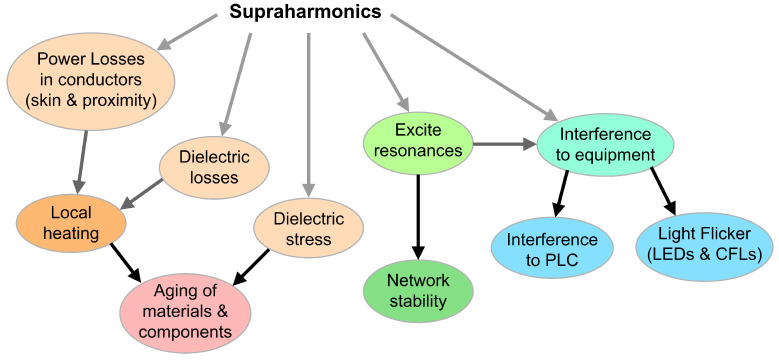
Overview of the negative SH effects showing the cause–effect relationship between them.

Such phenomena are depicted in Figure 1 and will now be analyzed in more detail in the following:

### 2.1. Power Losses

The basic rationale for power losses is related to resistive components in conductors that increase in value roughly proportionally to the square root of frequency as a consequence of the skin effect. If conducting materials are ferromagnetic, then the phenomenon is more evident (the skin depth being inversely proportional to the magnetic permeability). In addition, losses may also occur due to the magnetic properties of the material and hysteresis phenomena, as is commonplace for transformers. When windings (and tightly packed conductors) are considered, a second effect affects the distribution of the current in the conductor’s cross-section, the proximity effect, also approximately proportional to frequency.

An increase in conductor losses due to frequency is known and stigmatized in the IEC 61869-14 standard [30] (Figure 14A.4) when testing instrument transformers (ITs) for thermal losses at AC (50 or 60 Hz) rather than at DC. For large conductors with diameter *D* ranging between 40 mm and 100 mm, additional losses with respect to an equivalent DC current in terms of rms are about 150% at 50 mm diameter and 270% at 100 mm diameter for Alu conductors, respectively, and 180% and 330% for Copper conductors. The estimate is conducted using the Levasseur formula, as follows:

(1) $$k=0.25+\sqrt[{6}]{0.75^{6}+{\left(\frac{D}{4\delta }\right)}^{6}}$$

with *D* the conductor diameter and δ the skin depth in the conductor material.

A more accurate evaluation of SH effects up to 100 kHz at frequencies higher than those characteristics of the mains and its harmonics [31]. This work takes into account the formulas of the IEC 60287-1-1 standard [14], separately providing the correction factors $Y_{S}$ for skin effect, $Y_{P}$ for proximity effect, and $Y_{SC}$ for the screen losses (applicable to MV cables).

(2) $$Y_{S}=\left\{\begin{array}{cc} X_{S}^{4}/\left(192+0.8X_{S}^{4}\right) & \;0<X_{S}\leq 2.8\\ -0.136-0.0177X_{S}+0.0563X_{S}^{2} & \;2.8<X_{S}\leq 3.8\\ 0.354X_{S}-0.733 & \;X_{S}>3.8 \end{array}\right.$$

with $X_{S}$ containing the frequency dependence.

IEC 60287-1-1 proposes two formulations of $Y_{P}$ for the case of two-core cables (or two single-core cables) and three-core cables (or three single-core cables). The two cases are indicated by a subscript “2” and “3”.

(3) $$\begin{array}{cc} \; & Y_{P,2}=2.9\;\frac{X_{P}^{4}}{192+0.8X_{P}^{4}}{\left(\frac{d_{c}}{s}\right)}^{2}\; \end{array}$$

(4) $$\begin{array}{cc} \; & Y_{P,3}=\frac{X_{P}^{4}}{192+0.8X_{P}^{4}}{\left(\frac{d_{c}}{s}\right)}^{2}\left[0.312{\left(\frac{d_{c}}{s}\right)}^{2}+\frac{1.18}{\frac{X_{P}^{4}}{192+0.8X_{P}^{4}}+0.27}\right] \end{array}$$

with $X_{P}$ containing the frequency dependence, $d_{c}$ is the conductor diameter, and *s* is the separation of conductor axes (so equal to the sum of the diameter and the gap).

The two “*Y*” quantities are based on the calculation of the corresponding “*X*” factors that in turn contain two “*k*” coefficients related to the shape of the conductors: $k_{S}$ is largely equal to unity for round solid and stranded conductors for both copper and aluminum; $k_{P}$ is also unity for solid conductors and takes the value 0.8 for stranded conductors.

It is worth noting that this formula is stated as valid for $X_{P}\leq 2.8$, and, as observed in [31], this does not fit many practical cases, especially for SH frequencies.

A similar but different “$Y_{P}$” quantity is thus proposed [31], taken from [32]:

(5) $$Y_{P,D}=\frac{m\;{\left(d_{c}/s\right)}^{2}G_{P}}{2-5{\left(d_{c}/s\right)}^{2}H_{P}/12}$$

where the two factors $G_{P}$ and $H_{P}$ are again expressed with different approximations at variable $X_{P}$, as conducted before for $X_{S}$, and *m* is a factor for the cable geometry.

(6) $$G_{P}=\left\{\begin{array}{cc} \;11X_{P}^{4}/\left(704+20X_{P}^{4}\right) & \;0<X_{P}\leq 2.8\\ \;-1.04+0.72X_{P}-0.08X_{P}^{2} & \;2.8<X_{P}\leq 3.8\\ \;X_{P}/\left(4\sqrt{2}\right)-0.125 & \;X_{P}>3.8 \end{array}\right.$$

(7) $$H_{P}=\left\{\begin{array}{cc} \;0.333\left(1+0.0283X_{P}^{4}\right)/\left(1+0.0042X_{P}^{4}\right) & \;0<X_{P}\leq 2.8\\ \;0.095+0.119X_{P}+0.0384X_{P}^{2} & \;2.8<X_{P}\leq 3.8\\ \;\left(2X_{P}-4.69\right)/\left(X_{P}-1.16\right) & \;X_{P}>3.8 \end{array}\right.$$

For the last quantity, $Y_{SC}$, that accounts for the effect of the cable screen, the IEC 60287-1-1 proposes a simplified approach, taking the average of the two other factors, $Y_{S}$ and $Y_{P}$ (simplified $Y_{SC}$ expression).

To conclude, the three “Y” factors are put together to determine the “new” resistance R(f) including dependence on frequency and geometry:

(8) $$R\left(f\right)=R_{DC}\left(1+Y_{S}+Y_{P}+Y_{SC}\right)$$

If the simplified expression for $Y_{SC}$ is considered, then (8) can be rewritten as follows:

(9) $$R\left(f\right)=R_{DC}\left(1+1.5\left(Y_{S}+Y_{P}\right)\right)$$

For the accuracy of the published methods to predict additional losses, Topolski et al. [33] provided a comprehensive overview and comparison of various formulas, unfortunately with the frequency interval limited to about 2 kHz. The reference formulation is the one achieved by means of Bessel functions and the selected formulations for the comparison are the Levasseur Equation (1) (that the authors refer to as a Schneider Electric application note) and the IEC 60287-1-1 formulation considered above.

The results for a single-strand and multi-strand cable show that the IEC 60287-1-1 formulation for the two skin and proximity effect terms is not so accurate and underestimates losses for frequencies above about 1 kHz. However, when the terms of skin and proximity effect are taken together (so that the sum $Y_{S}+Y_{P}$ is evaluated), the correspondence with the theoretical reference method based on Bessel functions is almost perfect (up to 2.5 kHz). In [31], the comparison was carried out against Finite Element Method (FEM) results, showing a very good correspondence (maximum error of about 6%) if $Y_{SC}$ is also accounted for.

The relevance of the SH components evaluated in [31] over the 2 to 100 kHz interval is confirmed with an increase in total resistance by a factor of 6.5–7, proportional to the square root of the frequency ratio (=100/2=50). The minimum ratio with respect to the fundamental is about 3.5.

Looking for a suitable weight of SH amplitudes with the objective of limiting the losses to the same amount as caused by harmonic components, we observe a factor inversely proportional to the square root of frequency, giving the already mentioned factor of 7 between 2 kHz and 100 kHz. Before proceeding, the different standpoints of the IEEE Std. C57.110 [34] is considered, which defines a quadratic function of the harmonic order as impacting eddy current losses of transformers, whereas other stray losses have an exponent of 0.8 or less on the harmonic order (that agrees somewhat with the “square root of frequency” behavior). The standard, in its Annex C, reports some considerations on the effect of distortion at higher frequency (such as in the SH interval), observing that skin effect becomes relevant and deviations from the postulated “$h^{2}$” behavior are significant. A reduction of 10% is then estimated already for the first 20 harmonics if skin effect is taken into account. This correction is, however, not sufficient enough in the SH interval to match the $\sqrt{f}$ behavior.

In general, the total loss increase due to supraharmonics for the observed amplitude values is in the order of 10–20% of the losses ascribed to the harmonics (up to 2 kHz). The present harmonic limits have the objective of limiting losses to a value that requires an over-rating of cables of about 5% [19], considering such an amount of harmonic losses acceptable. This ratio is bound to increase with the extensive use of static power conversion units with increasing switching frequency (renewables, EV chargers, etc.) shifting distortion to higher frequency, namely reducing the harmonic contribution in favor of increased SH components.

Proposing an SH limit curve originating from a $1/\sqrt{f}$ weight starting from the harmonic limits for residential and industrial environments would imply additional losses equal to those allowed by present limits in the harmonic interval. Going down to a limit value for the single SH component is then more difficult if the number of SH components is not known a priori. A conservative standpoint may be assumed by adopting a sufficiently large resolution bandwidth (e.g., the frequently adopted 2 kHz), dividing the SH interval into 74 sub-intervals evenly distributed between 2 and 150 kHz with steps of 2 kHz. The risk is, of course, an excessive penalization, considering risky and non-compliant situations that see an uneven distribution of components, as is often the case.

### 2.2. Aging of Insulating Materials

#### 2.2.1. Partial Discharges

Partial Discharges (PDs) are a significant phenomenon affecting equipment and components and compromising their electrical insulation for which early detection of insulation failures and understanding of aging mechanisms is important for preserving continuity of service and avoiding more catastrophic spreads of failures [35].

Harmonic distortion is recognized as a PD contributing factor, as discussed in [15]. Although Total Harmonic Distortion (THD) is used as a parameter, it must be recognized that in special cases, the phase relationship between the harmonics and the fundamental can lead to very different peak values, as can be exemplified by considering a pure 3rd-harmonic distortion in-phase and out-of-phase with respect to the fundamental. In general, the effect of SH distortion on PDs is twofold: premature PD inception and quicker degradation of the material, e.g., as caused by self heating, combining with ongoing PD events.

One evident parameter is that the number of PD events per second *N* and the application of an overlapping supraharmonic component has been shown to increase it by approximately 10%, slightly more at the lowest frequency values (2 kHz to 5 kHz) than at the highest frequency components (6 kHz to 9 kHz) tested in [16,17]. The amplitude of the applied SH test signal was 10% of the fundamental.

A more detailed analysis and explanation is provided in [36]. Starting from the basic equation quantifying the critical electric field value of a cavity $E_{inc}$, they combine the presence of electric field values at the center of the cavity, equal to or larger than $E_{inc}$ and the availability of free electrons to trigger the discharge. A distinction is made for shallow-trapped charges (available for de-trapping in lower critical fields), moving then to deeper potential profiles as time passes (thus more difficult to start an avalanche discharge).The dependence on electric field strength, the probability of de-trapping (also in the first place depending on the electric field intensity), and the availability of “easy shallow charges” with the passing of time are all combined into an equation provided in [36] and reported below for a matter of convenience:

(10) $$N\left(t\right)=N_{0}\left(t\right)\left|\frac{E(t_{PD})}{E_{inc}}\right|\exp\left\{-(t-t_{PD})/\tau \right\}\exp\left\{|\frac{E(t)}{E_{0}}|\right\}+M\frac{dE}{dt}$$

where N(t) is the overall number of PD events through time, $N_{0}\left(t\right)$ is the number of PD events caused by the fundamental voltage only, $E_{inc}$ is the critical electric field value, τ is the electron availability decay time constant, *t* is time, and $t_{PD}$ is the instant of occurrence of the last PD event. The rightmost term represents the corrective factor for the effect of high-frequency oscillations, following the time derivative of the electric field E(t) in the cavity, and the constant *M*, adjusted experimentally, indicates the maximum availability of electrons during the supraharmonic oscillation.

Experiments made with superposition of SH components show that the long multiplicative term in (10) is not able to correctly predict the observed number of PD events, having focused on those scenarios where the positive half-cycle of the SH high-frequency oscillation increases the instantaneous electric field intensity. In reality, the effect of the fast rise of the electric field intensity must also be included, adding a term M(dE/dt) that depends on the time derivative of the voltage and is trimmed based on experimental results.

The conclusion seems to be that the influence on PD events is limited and reduces with frequency. The applied 10% of SH signal amplitude is already on the safe side for what are the usual amplitudes of SH emissions, so results can be assumed conservative.

#### 2.2.2. Degradation of Insulating Materials

Self heating is the most significant cause of the degradation of dielectrics and was demonstrated to be roughly proportional to ${\mathrm{THD}}^{2}$ and linearly to the harmonic order [15]. However, a non-linear phenomenon was observed in which, for a moderately large electric field (1 kV/mm) under sinusoidal voltage conditions, the current shows visible distortion. As a consequence, losses under the same amount of applied distortion are four times larger at 1 kV/mm than at 0.75 kV/mm, almost doubling heat losses with respect to the expected square dependency on electric field intensity.

Applied harmonic distortion levels show that THD values of 5% do not produce appreciable increases in heat losses, whatever the order of the harmonics (harmonic order between 3 and 17). A slight increase may be observed at higher THD values when the harmonic order is increased, and this may be similarly presumed once extrapolated to the SH interval.

Although the tests in [15] were carried out at harmonic frequencies (up to 2.5 kHz), it is a remarkable combined effect of distortion and electric field intensity, for which amplification of losses occurs at moderately large electric field intensity, still compatible with the design and use in normal operating conditions of components such as capacitors and cables.

Accelerated aging tests of insulating materials are reported in [17]: the tested materials are XLPE and alumina-loaded XLPE (with weight % ranging from 1% to 5%) and applied electric field intensity of several tens of kV/mm. It is interesting to observe that for moderate-to-high stress voltage levels (approximately 55 to 65 kV/mm), the effect of SH components is minor. Instead, when the stress level at the fundamental is set to a very high value (about 70 kV/mm), small values of a few percent make the difference.

It may be concluded that high-voltage stress, being one of the necessary ingredients for insulating material degradation by effect of SHs, occurs only in MV grids, and in particular conditions of high exploitation of the insulating material, such as at cable terminal joints and at very high MV levels (see, e.g., Section 2.4).

### 2.3. Aging of Capacitors

There are numerous devices with the dielectric exposed to some type of SH distortion. First of all, such devices encompass capacitors and supercapacitors, then, for example, batteries and general insulating materials.Insulating materials (already discussed above) are, in reality, exposed to SH voltage distortion (i.e., modulation of the electric field within the insulating material), whereas here, devices carrying significant SH current are considered (namely devices for storage and filtering). The focus is on those devices that are connected directly to the AC grid (e.g., for filters and power factor correction), whereas batteries and supercapacitors are connected instead through interface converters and are affected by the local converter ripple rather than by a grid-wide phenomenon. They are instead possibly directly connected when considering DC grids, and in this case, aging and stress consequential to distortion were discussed in [18].

In the harmonic interval, limits provided by capacitor manufacturers address the overheating caused by dielectric losses, and, depending on the application, an oversize of 10% to 20% compared to the dissipation at the fundamental may be sufficient.

Dielectric losses increase as well beyond the harmonic frequency interval, but a second dissipation mechanism that is usually negligible up to a few kHz takes place. Wiring losses are particularly evident in large capacitors that may be built around smaller modules with complex internal wiring, including fuses and inductive elements for protection [37]. Wiring losses follow what was stated in Section 2.1, with a contribution of skin and proximity effects.

### 2.4. Damage to MV Cable Terminations

Aging and failure of MV cable terminations is caused by localized heat possibly combined with worsened PD occurrence. Localized heat, in particular, has been observed in resistive stress-grading elements, where SHs affect the electric field distribution, causing hot spots [20]. The large intensity of the flowing current is due to the capacitive current being proportionally higher for increasing frequency.

A catastrophic failure (that represents a field evidence) occurred at the Eagle Pass substation [38] and in successive laboratory tests at ABB, Sweden, also reported in the same reference [38]. The measured on-site voltage distortion was distributed among some characteristic SH components with significantly large amplitude when compared to the 17.9 kV nominal value. The SH components were 1.28 kHz with 4.6 kV amplitude, 3.78 kHz (the 3rd harmonic of the switching fundamental) with 1.2 kV amplitude, and an atypical value of 12.4 kHz with 3.0 kV amplitude, due to network resonance. Relevant voltage distortion components around the 12.4 kHz resonance are, in reality, more than one, with the two adjacent to the resonance peak showing an amplitude of 2 kV and 1.2 kV, leading to an overall rms value of 3.8 kV.

During lab tests, it was determined that when using the worst-observed SH voltage levels, the heat caused at 12.4 kHz was 20 times higher than at 1.28 kHz. This is reasonable, as the electric field was approximately three times larger (due to a different spatial distribution estimated by Finite Element Method simulations) and the capacitive current approximately ten times larger (following the frequency ratio).

The mitigation adopted was a different type of cable termination and addition of a filter to reduce the 12.4 kHz component, which was caused by the resonance.

An approach is proposed in [20] that starts from the determination of the heat *Q*, dissipated in the stress-grading area and in the dielectric of the cable joint.This quantity *Q* is proportional to the frequency and to the square of the voltage, as it is put in relationship to the electric field intensity. Any temperature increase in the cable joint is shown to be proportional to the excess heat caused by the supraharmonics. For this reason, the $Q_{pu}$ quantity is calculated, separating and weighting the SH excess heat with that at the nominal frequency. The weighted ${\mathrm{rms}}^{2}$ of the SHs, each with amplitude $V_{h}$ multiplied by frequency, is divided by the weighted ${\mathrm{rms}}^{2}$ of the fundamental (i.e., $V_{1}^{2}$ multiplied by the fundamental frequency $f_{1}$). It may be said that the weighting with frequency accounts for the capacitive nature of the current, increasing with frequency. For a single SH component of frequency $f_{sh}$ and amplitude $V_{sh}$, the resulting *Q* value is:

(11) $$Q_{pu}=\frac{f_{sh}V_{sh}^{2}}{f_{1}V_{1}^{2}}$$

When $Q_{pu}$ approaches a value of 20, failure is very likely, based on evidence from the site and lab of [38], but subject to significant uncertainty, as the Eagle Pass cable was a unique case and the cable might have been defective or particularly susceptible to thermal stress.Some safety margins are then proposed [20], consisting of a multiplying factor *m* that takes values of 0.25, 0.5, or 1, identifying a green (m<0.25), yellow (0.25<m<0.5), orange (0.5<m<1), and red (m>1) area (the equal signs were omitted and assigning them to the lower or upper interval does not represent an issue).

A further simplification shown in [20] is to consider supraharmonics below and above 20 kHz, providing two straight limits rather than a more complex curve. Maybe this approach is oversimplied since the capacitive current is proportional to frequency and between 2 and 20 kHz there is a decade of frequency, and similarly, 20 kHz and 150 kHz differ by a factor of 7.5. In addition, the distribution of the electric field at termination was quite variable depending on frequency as well. For both factors, the variability is much larger than the trimming operated by *m*, so a more accurate frequency discrimination seems necessary.

A weighted sum, as commonly conducted for the weighted Total Harmonic Distortion index, could be adopted to provide an equivalent representation of the heat stress caused by a complex pattern of SH components. In the end, what is relevant is the heat stress that may be assumed proportional to the frequency. Since the SH spectrum may be characterized by a mix of narrowband and broadband components, aggregation with a large resolution bandwidth (e.g., 2 kHz) is more robust and the result less exposed to changes in the lateral bands of switching groups and other modulation byproducts. The examples in [20] indicate a risk of cable termination failure for single SH components in the order of 3% to 8%, meaning that for an SH spectrum populated by *n* components of not-too-different frequency, the limit must be reduced by $\sqrt{n}$, and with different frequencies for the mentioned components, each must receive a limit that is proportional to $1/\sqrt{f}$.

### 2.5. Interference with Equipment

This and the next subsection distinguish the two cases of interference with equipment in general and to PLC systems, in particular.

It is observed that the focus is on the SH frequency interval, with disturbance phenomena conducted at a higher frequency duly taken care of by the normative.They are the so-called “radio-frequency conducted disturbance”, occurring between 150 kHz and 30 MHz, and are extensively covered in terms of limits, test levels, and methods in a large set of standards, such as CISPR- and CENELEC-derived publications [39,40], which became the basic standards for emissions. On the other hand, there is another consolidated set of standards [41,42,43] for immunity of residential and industrial environments, and finally, the CISPR 16 series covers measuring methods and apparatus requirements and performance [44,45].

The technical justification for focusing on SH phenomena lies in the larger amplitude of SH emissions, propagating farther in distribution grids and much less attenuated than radio frequency signals by the EMI filters of equipment. In addition, local resonances in distribution grids are likely to occur at frequencies in the SH range due to a question of physical length and typical values of the components involved, as well as lower losses and therefore attenuations compared to the radio frequency range.

In general, due to the so-called “secondary emissions”, amplification of normal emissions is expected, as caused by the presence of other loads connected to the same grid. The reason is that the feeding impedance may be altered by the presence of, e.g., input filters and capacitive components, with consequential amplification of current emissions at some frequencies in the SH interval.

This was tested experimentally by combining, in pairs, an electrical vehicle, an LED lamp, and a TV set in [46]. The amount of measured emissions from the EV was in the order of 5 mA to 10 mA below 10 kHz and 5 mA located at an isolated component at 55 kHz. Theoretically, this was also demonstrated in [12,47] by considering a grid feeding a set of identical EVs located at different distances from the PCC on the same or two different branches of the LV grid. The EV was modeled using an experimentally determined curve of input impedance, and the mutual effect between EVs caused amplification of current emissions as soon as they were connected to not-too-far feeding points (in the order of 20 m to 50 m).

For comparison, EN 50627 [48] reports various cases of measured emissions from a wide range of sources, including portable tools, electrical appliances, and inverters (such as active infeed inverters, AICs). Low-power devices (such as LED lamps, tools, and various power supplies (e.g., for a fiber switch, a computer, and a surveillance camera) are in any case able to cause emissions in the order of 90 dBμV to 100 dBμV in the frequency interval 9 kHz to 70 kHz when measured with a standardized Line Impedance Stabilization Network (LISN). The equivalent current emission level may be obtained considering an LISN impedance variable between 5 Ω and 15 Ω, leading to a range of about 5 mA to 20 mA for the largest emissions. This is in agreement with [46] and provides an indication of worst-case emissions from low-power devices.

Inverters interfacing with renewables and energy storage devices are responsible in general for higher emissions, located as narrowband components at the switching frequency harmonics, usually with a fundamental in the 10 kHz to 20 kHz range. The examples of EN 50627 [48] report fundamentals of 16 and 18 kHz and the three PV inverters of Figure 20 of EN 50669 [49] have fundamentals of 15, 16, and 20 kHz. Except for dramatic cases, as reported for Belgium in the EN 50627, usual emission levels are about 20 dB higher than the previous ones, thus implying ten times higher current emissions and significant chances of interference.

These types of emissions have been observed in normal domestic LV grids, as electrical appliances, EVs, and PV inverters are commonplace in residential grids. The maximum values of emissions were in the order of 0.3 V to 1 V and up to 20 kHz, then gradually decreasing by no more than an order of magnitude.

Another way interference manifests is in audible noise [27], affecting electrical appliances (such as induction stoves and bell transformers), as well as EVs as victims of their own emissions. In the latter case, in fact, several similar or identical EVs were charging simultaneously and audible noise arose as a beating frequency of slightly different switching frequencies [28]. Furthermore, office equipment, such as computers and displays, is exposed to such problems because of the many systems employing static power conversion (e.g., Uninterruptible Power Supplies and power drives) with switching frequencies in the audio frequency interval [50,51].

The EN 61000-4-19 [52] has the objective of establishing relevant and reliable immunity test levels for the differential-mode disturbance in the SH interval. Unfortunately, this standard has not been applied yet, nor enforced by inclusion in any product or generic EMC standard. The first three test profiles (Level 1, Level 2, and Level 3) have amplitudes of 0.5, 3, and 12 V, respectively, set up to 9 kHz and then decreasing linearly in a log scale by a factor of 5, reaching values of 0.1, 0.6, and 2.4 V, respectively, at 95 kHz, where they are then held constant up to 150 kHz.

A straightforward comparison of the emission levels of PLC devices will be conducted at the end of this section. It is clear that in light of aggregation from different sources, as well as some margin of variability due to, e.g., grid resonances and slightly different product characteristics, Level 1 does not provide any confidence that a tested equipment will be immune to SH disturbance. Level 2 provides a safety margin of 10 dB to 15 dB with respect to the observed values, barely sufficient to cover the said aggregation. In fact, 10 sources disturbing the same frequency band within about 50 m would aggregate with a factor of 3.2 minimum, as resulting from a $\sqrt{10}$ assumption. This was demonstrated for harmonic and SH emissions of EVs in [53]. Level 3 thus seems highly advisable for all grids, noting that there is no longer any big distinction between residential, light industrial, and industrial environments regarding disturbance in the SH interval.

Some cases of highly disturbing inverters should instead be mitigated at the source, providing the necessary decoupling, e.g., by means of series reactors, thus implementing a so-called LCL filter.

### 2.6. Interference with Lighting Devices and Flicker

Flicker is considered here as photometric flicker; that is, a variation in the light output from a lighting device, such as LED (Light Emitting Diode) or fluorescent lamps, the latter often identified as CFL (Compact Fluorescent Lamps). Incandescent light bulbs are exposed to direct fluctuations in the amplitude of the mains fundamental, hence the main definition of light flicker in the standards, but lighting fixtures equipped with electronic conversion systems are instead potentially susceptible to high-frequency disturbance and can demodulate at low frequency, causing flicker.

Although in principle lighting devices are covered by EN 55014-2 standard [54] for immunity, such standards applied testing only for common-mode disturbance, using a 3 V test level suitable for residential/light-industrial environments. In reality, this standard and others do not consider differential-mode disturbance, as it is emerging from the SH distortion reviewed so far and does not ensure undisturbed operation in real usage scenarios. Modern lighting devices indeed have been shown to be susceptible to SH distortion. In [27], some cases of flicker interference are reported where SHs are suspected to be the culprit, such as in the case of a charging Tesla EV [55].

Regarding the susceptibility of a lighting device to a specific disturbance in terms of frequency spectrum and intensity, it is, in general, difficult to establish a rule as different lighting devices react differently, as demonstrated, for example, in [56], where two LED devices were quite insensitive to two disturbing test signals, whereas the third device showed a consistent reduction in luminosity by 40% to 50%, and the fourth and fifth reacted with a significant modulation of light output in the order of 5 lux (out of an average of about 400 lux) and a modulation of the input current of 5 mA (out of about 55 mA). The repetition frequency of the flicker disturbance is in the order of 0.1 Hz, so not in the interval of maximum nuisance, which is located around 8 Hz but still perceivable.

The applied disturbance test signals were two realistic signals recorded on site, showing two different spectral behaviors: distinct spectral components in the range of 1 kHz to 10 kHz with harmonics of lower amplitude extending up to 50 kHz, and one with spectral occupation of 40 kHz to 60 kHz, both with the largest components at about 4 V and 2.5 V, respectively.

In [57], a more extended test of LED and CFL devices was conducted (among other home appliances) by applying immunity test signals in compliance with EN 61000-4-19 [52]. The applied test level was “level 3”, so 12 V up to 9 kHz, from 12 V to 2.4 V decreasing logarithmically between 9 kHz and 95 kHz, and then the amplitude remained at 2.4 V until 150 kHz. Apart from flicker, the other negative effects were a temperature increase and in one case (with the lamp power supply based on a capacitive divider), audible noise. In all cases where the input impedance of the device could reduce at some frequency in the SH range, the sharp increase in current was accompanied by disturbances such as audible noise due to, e.g., mechanical resonance of inductors. The current increase is also responsible for power losses (from which the temperature increased), particularly evident for devices with a capacitor on the utility side.

Summing up the results of the immunity tests on a wide range of home appliances (half of which are LED or CFL lamps), the application of the EN 61000-4-19 immunity test signals caused audible noise in 45% of the cases, whereas flicker was caused in 10% of the cases, with another 45% of the devices found to be immune.However, the dependency on the test frequency is not clear, as series resonances of the subjected devices were observed at low SH frequency, whereas above 50 kHz, the devices seemed quite immune. It is also observed that the Level 3 test amplitude is quite similar to that of the two realistic test signals used above, exceeding at low frequency by a factor of 3.

One example of complained flicker interference from an EV is reported in [27], providing measurements of the EV disturbance; unfortunately, in terms of current, i.e., about 20 mA to 30 mA in the intervals 1 kHz to 10 kHz and 45 kHz to 50 kHz, they cannot be compared directly to the other test signals expressed in terms of voltage.Considering the impedance values discussed in Section 3.2 of [53], these current values translate into voltage values in the order of 100 mV to 200 mV, so an order of magnitude less than the test voltage amplitudes. However, a complaint has been the appearance of flickering, which can be explained by the large intensity fluctuation observed at around 40 Hz.

### 2.7. Interference with PLC

Apart from some episodes of interference caused by PLC systems (e.g., to lighting, as cited in Section 5.2.1 of EN 50627 [48]), PLC technology is in general the victim of various forms of emissions. Emissions in the SH frequency interval are not generally disciplined by applicable limits and, even for those products with an emission standard (e.g., lighting and electrical appliances with EN 55014-1 [58]), there are episodes of non-complying devices. It is observed that the limits of EN 55014-1 in the first 9 kHz to 50 kHz range are quite high, allowing 110 dBμV of quasi-peak amplitude.

Emission measurements in the SH interval carried out before 2010 seemed to lead to the conclusion that interference with PLC was unlikely, as observed in [59]. However, for successive years, various episodes of interference with PLC devices were described in EN 50627 [48], mainly based on documented investigations carried out in Sweden by the local authority. The interfering levels at various frequencies are confirmed to be in excess of 100 dBμV, and interference was suppressed with the application of mitigations (namely EMC filters), bringing emissions to a safe lower level. These two concomitant amplitude values for selected components (with and without interference) for the same system under the same conditions are extremely useful for defining a reference line of interference below the lowest of such interfering cases.

PLC systems are mainly exposed to in-band interference, where emissions happen to occur in their operating bands. The PLC operating bands may vary largely for extension and location over the frequency interval, as shown in Table 1, but all share a significant portion of the SH interval.

A study of the various apparatuses connected to a microgrid (PV inverters, turbines, pumps, and battery chargers) [60] has shown that the PRIME PLC system (an Orthogonal Frequency-Division Multiplexing, OFDM, narrowband device) is significantly disturbed by narrowband components originating from the battery charger and falling into the PLC operating band, between approximately 40 and 80 kHz: the relevant disturbing components are 104 dBμV at 48 kHz and 81.3 dBμV at 72 kHz. It is curious that two narrowband components can disturb a system that in principle should be able to hop on different frequencies and tolerate momentary interference on some of them (if not to exclude such disturbed frequencies from the list of the OFDM ones).

**Figure 2:**
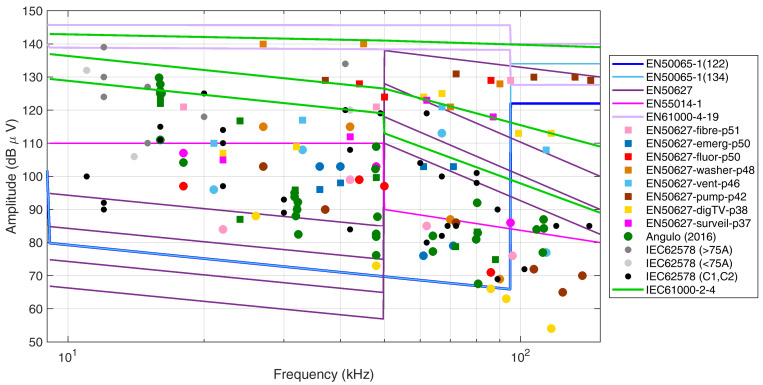
Overview of emission levels: (blue and light blue lines) limits of emissions from EN 50065-1 for PLCs of categories 122 and 134; (purple lines) limits of emissions from EN 50627 for various classes of converters; (magenta line) limits of emissions from EN 55014-1; (pink lines) immunity test levels prescribed by the EN 61000-4-19 (levels 3 and 4); (green curves) IEC 61000-2-4 compatibility levels for classes “1 + 2a”, “2b”, and “3” from bottom to top; (gray/black circles) AIC emissions data taken from IEC 62578 for various categories (>75 A, <75 A, and “C1 and C2”); (green circles) emissions of various PV inverters taken from Uribe-Perez et al. [60]; (squares) confirmed PLC interference levels, reduced ones after mitigation (circle), with colors indicating various sources (detailed in the legend).

The various emission cases discussed so far are collectively represented in Figure 2, showing also the cases of levels with confirmed interference with PLC devices.

### 2.8. Interference with Energy Meters and Residual Current Devices

Energy meters (or smart energy meters) and residual current devices (RCDs) share components that make their susceptibility to waveform distortion similar for at least two characteristics: the large crest factor, as caused by low-order harmonics and artificially induced by adding a large capacitor in [61], bringing current sensing electronics into saturation; the behavior of RCDs to high-frequency superposed components is more complex, summing up the effects on the differential transformer and detector [62], ending in both unwanted tripping and desensitization, impacting availability and safety, respectively.

Interference with energy meters may be split into two parts: the interference of the distorted waveforms to the metering function, i.e., the correct measurement and then calculation of active and reactive power terms, and the interference with the communication function of smart energy meters; that is, based on PLC technology, which has already been discussed in the previous Section 2.7.

The first point was studied by applying low-frequency distortion and considering how the algorithm incorporated into the energy meter evaluates the contribution of harmonic terms. Interference with the metering function has been intensively investigated in the last twenty years, providing a knowledge set regarding the impact on the metering accuracy and the relevant levels of distortion [63,64].

In [61], the analysis of the resulting interference caused by LED + CFL lamp load shows that the rate of rise of the current is the relevant factor. Changing the dimming angle, the rate of rise increases by more than an order of magnitude up to 1.1 A/μs, with peaks of the input current up to about 50 A. Strictly speaking, such impulsive disturbance is not exactly an SH phenomenon, but the spectrum occupation is in the SH interval. The tested static meters using a Rogowski or Hall effect sensor for the measurement of the current show, as a consequence, a wide change in the read energy, almost surely caused by saturation of the post-processing electronic circuits. In particular, the Rogowski-based meters show a very large positive change (about +300%), whereas the Hall-based meters show a moderately large negative change (−50%); those using a shunt have almost no change in their output.

A complex scheme of interference has been observed also for residual current devices (RCDs) [65] with different responses depending on models. Apart from undesirable tripping by superposition of SH components, what is most relevant is the possible RCD desensitization, tripping then at higher fundamental (50 or 60 Hz) current values than required for electrical safety. Tests performed in [65] at significant, but not unlikely, injected SH current components (800 mA between 1 and 5 kHz) confirmed a desensitization of 2 to 2.5 for two different RCDs. Tripping occurred at residual current values of 58 mA and 76 mA at 50 Hz rather than the set 30 mA. Such current values under the usual assumption of a 5 Ω grid impedance (the LISN value) correspond to 4 V of SH voltage distortion. By extrapolation of the two SH disturbance levels (800 mA and 1300 mA) for the tested RCD model (“A-30”), the minimum SH current causing a deviation above the desired 30 mA tripping threshold is about 600 mA, which corresponds to about 3 V or 130 dBμV at low frequency (approximately over 2 kHz to 10 kHz). The assumed 5 Ω value of grid impedance surely represents an overestimation if compared, for example, with the impedance curves shown in Figure 18 of IEC 62578 [37], lying in the 0.5 Ω to 4 Ω interval. Such an overestimation provides a margin when calculating voltage distortion from an observed current, but not the opposite, and we have seen that current, rather than voltage, is often the relevant disturbing quantity.

There are also episodes of SH interference with leakage monitors, manufactured for a wide operating interval for AC and DC power distribution, including pulsed DC waveforms as Type B devices, but failing to operate selectively on DC in the presence of high-frequency pollution. This is behind the recent availability of models, such as the Bender MRCDB300 [66], with a selectable low-pass function, also in response to the update of the IEC 60755 standard, indicating a required response to residual smooth DC current, independent of the speed of application (suddenly applied or slowly rising) [67].

### 2.9. Sh Transfer Efficiency between MV and LV Levels

The negative effects discussed above can be seen as unique to the MV or LV levels when the physical phenomena and devices involved are considered with a closer look. Interference with PLC equipment and systems is likely to occur at the LV level, whereas cable termination is thought to be characteristic of MV networks, as is the aging of insulating materials, particularly in the case of pre-existing highly fundamental voltage stresses.Conversely, it can be observed that dielectric aging occurs equally at LV and MT levels, as filters are used indiscriminately in both cases.

Resultingly, what is relevant for liaising the commented SH distortion levels and deriving comprehensive acceptance limits is the quantification of the transfer between the MV and the LV sides of distribution transformers at SH frequencies.The SH frequency interval is such that the relevant coupling between the primary and secondary windings is a mix of inductive terms (designed magnetic coupling between windings operating at the fundamental frequency and above it) and capacitive terms (mutual stray capacitance).

A few publications [68,69] provide some measured curves of the transfer ratio between the LV and MV sides and between phases. Usually, such a transfer ratio is expressed by a multiplication coefficient of the nominal transfer ratio to the fundamental: it is thus expected around unity at low frequencies, then decreasing at high frequencies, except in the case of resonances.

The behavior of a small 100 kVA, 20/0.4 kV (50:1 nominal ratio), delta-wye-connected transformer was studied in [68]. The transfer ratios from LV to MV were measured first (test named “P1”, transfer ratios $k_{11}$, $k_{12}$, $k_{13}$), with *k* indicating LV to MV and “11” used for the same winding at LV and MV, respectively, “12” and “13” for the coupling to the other two windings), and then applying the test signal on the MV side; the opposite MV-to-LV transfer capability was measured (test “P2”, transfer ratios $h_{11}$, $h_{12}$, $h_{13}$), with *h* indicating MV to LV and the numeric indexes having the same meaning). We can conclude the following:

- The transfer between LV and MV sides undergoes a significant resonance that is not visible from the MV side; it is noted that such 10× resonance, including the 50:1 nominal ratio, would peak at 500× of voltage on the MV side. That is hard to believe; apart from this, the values are low, being between about 0.2 and 0.01;
- The usual transfer behavior is that the phase L1 on the LV side influences the corresponding phase L1 on the MV side, and similarly, phase L2 but not phase L3; for a delta-wye transformer, this is customarily used for MV to LV distribution;
- The transformer has a symmetric behavior for which the self-transfer ratios (each LV phase to the corresponding one on the MV side) is the same (Figure 8 of [68]);
- The transfer from MV to LV for the same phase is more effective and does not show significant variations vs. frequency, being at around unity (ranging between 0.64 and 2.25 up to 80 kHz).

The more recent study in [69] tested a 1 MVA, 10.5/0.4 kV, delta-wye-connected transformer for both current- and voltage-transfer characteristics. The frequency interval is unfortunately limited to below 8 kHz, thus limiting the generality of the results that occur around the transformer resonance at some kHz (as identified in [68]), with the risk of being pessimistic. The reported results may be synthesized as follows:

- Regarding the current LV-to-MV transfer, the results for L1 to L1 show an almost unity transfer ratio between 1.6 kHz and 7.6 kHz, with a slight amplification (30%) at some components;
- Voltage (for L1 to L1 from LV to MV) is attenuated by a factor of 3 to 10 in the same frequency range, except above 6.7 kHz where the ratio is almost unity; this appears from Figure 4 of [69], where the values seem to be reported without including the 26:1 transformer ratio, but the considerations of the authors lead to the conclusion that the ratio was already included but not annotated;
- For the MV to LV transfer, the results provided by [68] are not fully confirmed, having found a slightly larger variation (more persistently around a factor of 2 to 3); what is relevant is that in the case of an unloaded transformer (not magnetized), the behavior is quite different and variable;
- Last, the LV-to-LV transfer occurring between the secondary windings of two different transformers through the MV grid was studied and the observed transfer ratio was more than unity (e.g., 2 to 3) at several frequency points, whereas some attenuation should, in general, be expected; this is a relevant result regarding the propagation of interference within the same LV grid, but on different feeders and parts of the grid.

## 3. Bibliometric Assessment of References and Findings

This short section aims to show the consistency, in bibliometric terms, of the literature search that provided the sources on which Section 2 and Section 4 are based. The bibliometric assessment is conducted by considering the number of search hits, their distribution through the years, and the coverage of the specific discussed phenomena.

The examined literature includes theses, white papers, and brochures from industry standards.A total of about 160 items were identified with various search keywords after the removal of duplicates. Keywords used to perform the search were selected from the following set: “supraharmonic”, “propagation” or “aggregation” or “summation”, “photovoltaic” (also “PV”) or “wind” or “electric vehicle”, and adding the terms “medium voltage” and “experimental” to narrow down the search and find some hits to supplement otherwise less-considered aspects. In addition, other sources were found by using the list of references in the first set of positive hits and also the review process helped to refine the search. The search process ended when all successive searches did not retrieve any new entries. The collected items were then subject to deeper examination to remove those not providing quantitative information related to the issues discussed in Section 2.

**Figure 3:**
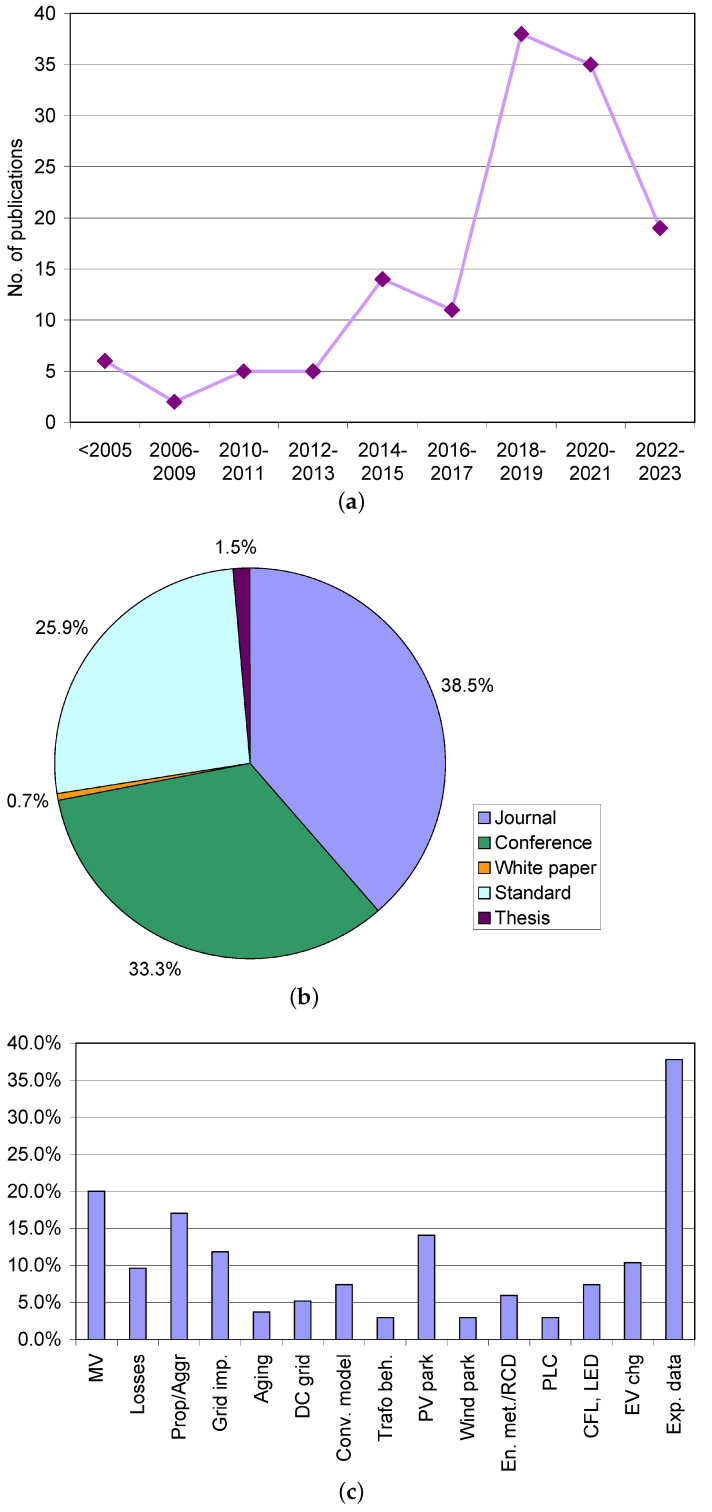
Bibliometric results: (**a**) number of sources through the years; (**b**) subdivision of sources per publication type; (**c**) shared percentage of prevalent keywords.

The remaining items were 100 and to these scientific references 35 relevant standards were added. Their distribution in terms of year of publication, type of publication, and characterizing keywords is shown in Figure 3. With a reduced database size, to avoid erratic fluctuations in publication years, in more recent years they have been paired and grouped by four between 2006 and 2009, or cumulatively, all comprehensively before 2005.

It may be noted that the number of publications on this subject has increased through the years, although recently, the number is reducing, probably because the contribution to the standardization activities has been somewhat reduced and the focus has been moved to the evaluation of spectra, the equivalence between algorithms and detectors, and related uncertainties (this part is briefly discussed in Section 4.1, but references are limited to a dozen). The activity in the newly EU-funded project ADMIT (https://www.admit-project.eu/, accessed on 7 April 2024) should bring new research impetus, applied to the measurement of SH phenomena with instrument transformers, in particular, in MV networks. The recorded number of contributions is in agreement with what is reported in [70], where a reduction for the years 2021 and 2022 is pointed out, although the absolute numbers in [70] are double due to the larger scope of the publication.

Regarding the type of publication, there are some special conferences on power quality and distribution grids (such as CIRED and CIGRE congress) that have attracted several publications; the fraction of journal papers is in any case significant, well positioned at one-third of all the references.

As for the keywords indicating the subtopics dealt with by the respective publications, there is good horizontal coverage of “losses”, “transformer behavior”, “aggregation”, “propagation”, and “aging” (which we have discussed in Section 2), all with about 5% of coverage. It is underlined that a keyword is considered dealt with if specific results and analysis are provided.

## 4. Lessons Learned and Compatibility Levels for the SH Interval

### 4.1. Existing Normative Limits and Compatibility Levels for the SH Interval

EMC and PQ standards may indicate limits of emission, levels of immunity, or reference compatibility levels for the SH frequency interval. First of all, a distinction must be made saying that we are focusing on differential-mode conducted phenomena, for which many EMC standards are irrelevant as they consider mainly common-mode disturbances.

Compatibility levels are defined as a reference level for the coordination of emission limits and immunity test levels for a given environment. They are also an indication of the expected level of disturbance in that environment. There is a set of “environment” EMC standards covering public networks at LV (EN 61000-2-2 [71]) and MV (EN 61000-2-12 [72]), as well as industrial networks (EN 61000-2-4 [73]). In reality, for the SH frequency interval, EN 61000-2-12 does not contain any indication limiting the scope to harmonic distortion. From the list of sources (arc furnaces, adjustable speed drives, …), it is also evident that twenty years ago, the impact on the grid of electric vehicles and renewables was not foreseen. The EN 61000-2-4 reports three environments, ‘1’ (or ‘2a’), ‘2b’, and ‘3’, the former corresponding to the residential/commercial LV environment of the EN 61000-2-2.

These levels were extensively discussed and compared in [53] for distortion caused by EVs during charging operations, considering harmonic and supraharmonic intervals. It is observed that two issues make the definitions of SH limits or compatibility levels more complex: first, the effect of the grid impedance and of resonances with variable factors of merit amplifying or attenuating emissions in terms of voltage and/or current [1,3,4,11]; second, when comparing values in the harmonic and supraharmonic ranges, the different resolution bandwidth and the narrowband or quasi-broadband behavior of components at different frequencies must be considered.

When EMC standards in a wide sense (including power quality standards, such as IEC 61000-4-7 [74] and IEC 61000-4-30 [75]) provide an indication of limits for conducted phenomena, low- and high-frequency and narrow- and broadband aspects have a significant influence on measurement and assessment methods. The following list exemplifies the most relevant factors that make a comparison between different standards difficult:

- Resolution bandwidth (RBW) is a relevant parameter, both when using a direct frequency-domain approach (e.g., a scanning receiver), or an indirect one, processing time-domain recordings by Discrete Fourier Transform. RBW values are usually standardized at 5 Hz [74], 200 Hz [45,74], 2 kHz [75], and 9 kHz [45]. The chosen RBW value has various implications:
  – On the aggregation of nearby spectral components, for which the individual compliant spectral components can be measured as higher-amplitude equivalents no longer conforming to the limits; it is reasonable that RBW values should be selected in agreement with the bandwidth of the victim; that is, power losses may be evaluated with large RBW values, whereas interference with PLC channels should be assessed with an RBW comparable to the channel width;
  – On the response to complex time-varying waveforms containing impulsive and oscillatory parts [76,77], for which a large RBW is able to track very fast signals, but loses resolution of the spectral representation;
  – On the accuracy of the amplitude estimate of components [77], at least simply for the contribution of the incoherent noise falling within the measuring bandwidth, apart from the composition of different adjacent spectral components with their phase and time relationship into an equivalent one.
- Apart from the influence of RBW on amplitude accuracy (as briefly discussed above), the intensity estimate for frequency-domain measurements is provided by amplitude detectors after demodulation during the scanning. Such detectors may be an rms detector, a peak detector, or a quasi-peak detector, the latter causing some concern. Its use is supported by CISPR and generally by RF EMC standards as an emissions weighting for a hypothetical jamming of analog radio transmissions for broadcasting purposes (no longer so widely used in the modern world of digital communication);its output being dependent on the time distribution of the spectral characteristics of the incoming signal, it can hardly be compared to a simpler rms or peak detector, as commented in [78] for impulsive emissions originating from pantograph electric arcs. Nonetheless, its application is still mandatory and has recently attracted a lot of effort to devise an efficient time-domain implementation [79,80].
- In general, the representation of time variability of disturbance in the short and long term is challenging for a matter of balancing accuracy and time granularity with the compactness of the representation. Standards IEC 61000-4-7 [74] and IEC 61000-4-30 [75] propose the aggregation of spectral components over intervals in the order of hundreds of ms (200 ms) and some seconds (3 s); IEC 61000-2-4 [73] states clearly that disturbance is assumed stationary over the 200 ms time interval. Longer aggregation times, such as 10-minute intervals or daily values, are required for the assessment of grid power quality and quantification of compatibility levels [71], but are not at all able to adequately represent spot scenarios of interference with victim equipment. This has two reasons: interference may take place in short time intervals and originate often from modulation byproducts with dynamics in the order of ms. An example of the latter is Figure 5 of [27], showing the pulsating spectrum of an EV input current causing flicker. If SH components are instead evaluated for relevance to human exposure to the electromagnetic field, the required averaging intervals are long, to evaluate impact in terms of thermal effects, as discussed in [13].

Emissions in the SH frequency interval are well covered for what concerns intentional (or in-band) and non-intentional (or out-of-band) emissions of PLC devices (standard EN 50065-1 [81]).

After the overall discussion of emissions and relevant parameters for their quantification, an underlying inconsistency must be underlined. Compatibility levels, as provided by EN 61000-2-4 [73] at its Tables 4 and 5 for 2 kHz to 9 kHz and 9 kHz to 150 kHz intervals, are clearly covering differential mode for the following reasons: they follow a quantification of THD, the former levels for the 2 kHz to 9 kHz interval are provided as a percentage of the fundamental, and explicit reference is made to IEC 61000-4-7 [74]. Instead, emission limits are always provided for common-mode disturbance only, in line with CISPR standards. Immunity test levels are commonly in common mode as well, but EN 61000-4-19 [52] addresses immunity to differential-mode disturbance. Equivalency between differential-mode and common-mode levels is not exact, but, based on observed grid impedance values at high frequency, we can say that in the higher portion of the 2 kHz to 150 kHz interval, differential-mode and common-mode impedance values are quite similar, although the latter slightly larger.

The focus in this work is on levels and limits of the differential mode, for which we can consider the EN 61000-2-4 compatibility levels, ranging between about 1 V for classes 1 and 2a to 10 V for class 3 in the 2 kHz to 50 kHz interval, and between about 0.1 V for classes 1 and 2a to 10 V for class 3 in the 50 kHz to 150 kHz interval. We will see that derived limits of Section 4.2 below are right in this range of values, implying the following:

- Environmental EMC standards (with notation 61000-2-X) should be updated and compatibility levels harmonized without dramatic changes;
- Present compatibility levels are such not to significantly penalize manufacturers of power conversion systems, either standalone or embedded (for a wide range of applications, such as EV charging, lighting, consumer electronics, etc.);
- If such environmental standards are duly considered normative references to limit emissions, adverse phenomena are under control; it goes without saying that EMC certification of products should be taken seriously, as well as verification of compliance once placed on the market.

### 4.2. Limits Based on Documented Negative Effects

As reviewed in Section 2, there are documented negative effects (or, in general, “interference”) for different types of phenomena that have different intensity levels and time dynamics. For example, losses and self-heating are slower and appear at higher intensity than EMI to equipment, and in particular, PLC devices.

In general, the influencing mechanisms are such that a reference level (or limit) that reduces with frequency may be expected; losses are proportional to the square root of frequency, induction is proportional to frequency, and capacitive current increases with frequency. For interference with PLC devices, as the transmission level is constant over the operating frequency, a constant reference level may be expected, although a slight reduction in the received signal may be considered for increasing frequency (as caused by line attenuation).

From the previous discussion, we may consider the following points:

- Documented interference with PLC devices, considering both cases of presence and absence of interference; to this aim, the results shown in Figure 2 are used; values for mains signaling at the MV level are not established, as pointed out in EN 61000-2-12 [72], still in the 2003 version;
- Losses and consequential heating taking the harmonic limits as reference for the residential and industrial applications, namely considering the current distortion limits of EN 61000-3-2 (2019) [82] and EN 61000-3-12 (2019) [83], respectively; with a general assumption regarding the expected grid impedance, such limits are transformed into voltage distortion levels and then compared to those of EN 61000-2-2 (2019) [71] and EN 61000-2-4 (2020) [73];
- Effects at MV level, considering the critical values impacting the reliability of cable joints (see Section 2.4);
- Interference with energy meters and residual current devices (see Section 2.8).

The other phenomena discussed in Section 2 were evidently relevant only at higher SH distortion levels and are thus not further analyzed.

#### 4.2.1. Interference with PLC Devices

**Figure 4:**
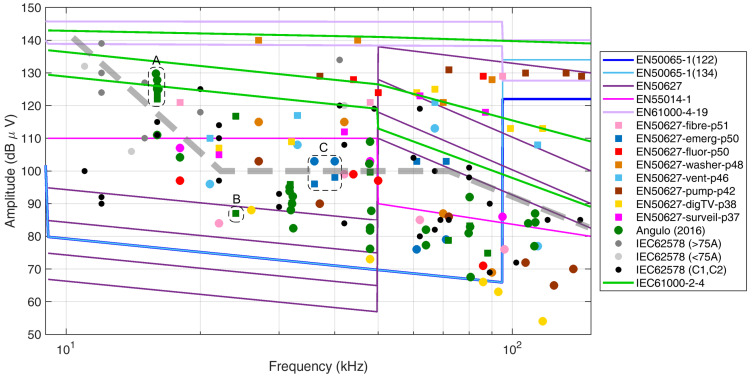
Details of the emission levels of Figure 2 with superposed proposed limit curve (dashed gray). Groups “A”, “B”, and “C” are discussed in the text.

Section 2.7 has discussed the problem of interference with PLC devices showing positive cases of interference as mostly reported in the standard EN 50627 [48]. Such levels, as shown previously in Figure 2, are reported in Figure 4 with overlaps of some annotations and a proposal for an overall reference level to be considered as a limit with respect to interference with PLCs alone.The purpose is to provide good coverage of the SH interval in terms of demonstrated interference cases, as well as lower levels in the same scenarios at which no interference occurred. The objective is attained when the “no interference” levels are sufficiently separated from the corresponding interference cases and also well leveled with respect to the corresponding “no interference” or “interference” cases at other frequency values nearby (so as to postulate homogeneous behavior at similar, but not the same, frequency values).

Three groups of points are highlighted by a dashed black circle and labeled as “A”, “B”, and “C”. Such points show some contradictory behavior with respect to the expected arrangement with the interference case (the square symbol) above the no interference case (the circle symbol):

- Group A: The points have a spread of 8 dB only, but with the interfering value reported as the lowest one; these values come from different PV inverters connected at the same grid, where interference was reported for one of the PLC devices in the same grid. It is thus possible that the attenuation from the source to the victim PLC is variable and accounts for some dB of variation, as well as that these values are not really interfering or not interfering with the PLC operation, as they fall outside the 42 kHz to 89 kHz Prime PLC operating band.
- Group B: similarly, this is an isolated point reported as interfering, but part of a broader spectrum where the interfering components fell inside the Prime PLC band.
- Group C: these two pairs at 35 kHz and 40 kHz are also very likely outside the operating band of the PLC in question, whereas confirmed interference for the square symbols is caused by the other points of the same case (blue squares) at 60 kHz and 70 kHz.

In Figure 4, a thick dashed gray line is proposed (as a limit of 100 dBμV above about 20 kHz with two variants with different slopes): this limit curve separates the circles from the squares, i.e., the “no interference” cases from the “interference” cases. The proposed limit of emission of 0.1 V provides a margin of more than an order of magnitude with respect to the Level 3 immunity levels of EN 61000-4-19 [52], already commented on in Section 2.6.

It is observed that such division is approximate, especially at a low frequency where the points are at the margin or outside the typical PLC band (in principle extended from 3 to 95 kHz and identified as a CENELEC “A band” in [60]).

In addition, it is possible to observe that higher levels may be allowed at lower frequencies, just moving the corner point slightly back to about 20 kHz and tilting the dashed limit curve (resulting in the other darker dash-dot profile). It can be noted that in the low-frequency part of the SH interval, between approximately 2 and 15 kHz, the variability of the proposed profile is significant and reference values here should be justified with other considerations, based on different approaches, such as extension of the harmonic limits for generic interference with equipment and limitation of losses, as well as other mechanisms of aging and deterioration (for example, for MV cable joints), both considered in the following.

#### 4.2.2. Losses and Self-Heating

This section contains a working example of the assignment of convenient SH current limits, starting from two assumptions. First, the harmonic limits for residential (≤16 A [82]) and industrial (≤75 A [83]) loads aim at limiting power losses and self heating; values are shown in Table 2, to divide then for the respective reference current values $I_{r,Res}$ and $I_{r,Ind}$ to obtain the limit in %. Second, the number of SH components is known and is assigned for three sub-bands of the SH interval, namely 2 kHz to 9 kHz (in this example, three components), 9 kHz to 50 kHz (in this example, three components) and 50 kHz to 150 kHz (in this example, six components). This assumption is in line with what is reported in [84] for a complex load such as the inductive charger of an electric bus.

A Total Supraharmonic Distortion (TSHD) index is considered similar to the classic Total Harmonic Distortion (THD); that is, corresponding to the root of the sum of the square of normalized component amplitudes. The use of subscript “I” indicates that the index refers to the current.

(12) $$THD_{I}=\sum_{h=2}^{h=40}{\left(\frac{I(h)}{I(1)}\right)}^{2}$$

(13) $$TSHD_{I}=\sum_{f=2\;kHz}^{f=150\;kHz}{\left(\frac{I(f)}{I(2\;kHz)}\right)}^{2}\;\;TSHD_{I}^{\prime }=\sum_{f=2\;kHz}^{f=150\;kHz}{\left(\frac{I(f)}{I(2\;kHz)}\right)}^{2}\sqrt{\frac{f}{2\;kHz}}$$

**Figure 5:**
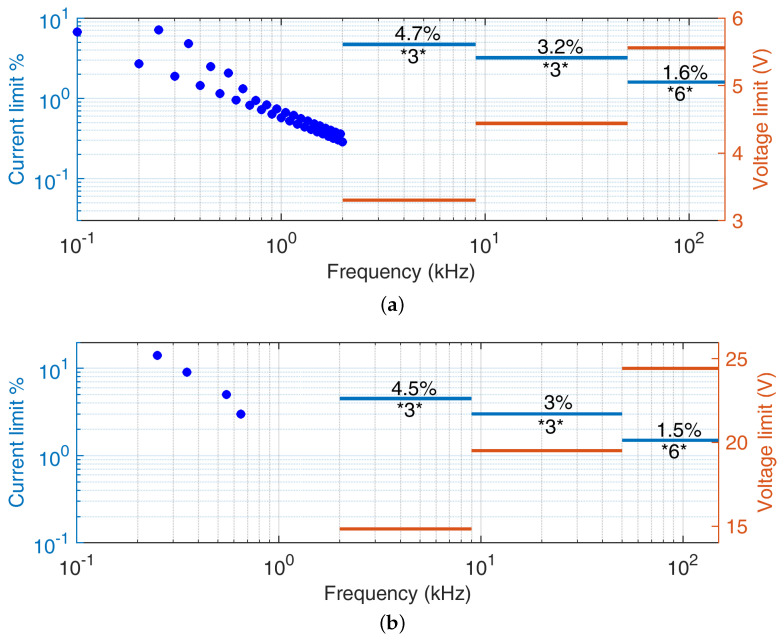
Example of SH limits for the three sub-bands (2 kHz to 9 kHz, 9 kHz to 50 kHz, and 50 kHz to 150 kHz): current (light blue) and derived voltage distortion value (light brown). On the left-hand side, the corresponding current limits for the harmonic interval are visible. (**a**) Residential environment (reference standard IEC 61000-3-2 [82], Class D equipment); (**b**) industrial environment (reference standard IEC 61000-3-12 [83]).

Each component is combined with the others of the same sub-band with a square-root-of-frequency criterion, trying to balance the partial contribution to the heat of each sub-band, and then the complete ${TSHD}_{I}$ is calculated. It is the same as the estimated $THD_{I}$, following the limit of the respective standards for residential or industrial applications. The industrial case is taken with short circuit ratio $R_{sc}=66$. The result is shown in Figure 5, having also estimated the allowed tolerable voltage distortion by taking the reference current $I_{r,Res}$ as 16 A and $I_{r,Ind}$ as 75 A, and having estimated the grid impedance using the curve of Figure 20 of the IEC 62578 standard [37]. The absolute impedance values are as follows: 3.7 Ω at 2 kHz, 5.22 Ω at 9 kHz, 14.41 Ω at 50 kHz, and 32.72 Ω at 150 kHz. The reference voltage values $V_{r,Res}$ and $V_{r,Ind}$ are taken as 230 V and 400 V, respectively.

The resulting SH limits in % are almost the same for the residential and industrial cases: this is due to the $THD_{I}$ limit value, used as a reference that is 15% and 16% in the two cases.

It is also remarked upon that for a larger number of components in each sub-band ($N_{SH-B1}$, $N_{SH-B2}$ and $N_{SH-B3}$), the corresponding limit must be reduced. The reduction factor ($k_{B1}$, $k_{B2}$ and $k_{B3}$, respectively,) would be proportional to the square root of this number compared to the studied case ($k_{B1}=\sqrt{N_{SH-B1}/3}$, $k_{B2}=\sqrt{N_{SH-B2}/3}$ and $k_{B3}=\sqrt{N_{SH-B3}/6}$). The assumption that the components are not synchronized and independent is not necessary, as the investigated effect is the power loss and consequential heating that follows by its own nature an rms summation.

#### 4.2.3. Stress of MV Cable Joints

**Figure 6:**
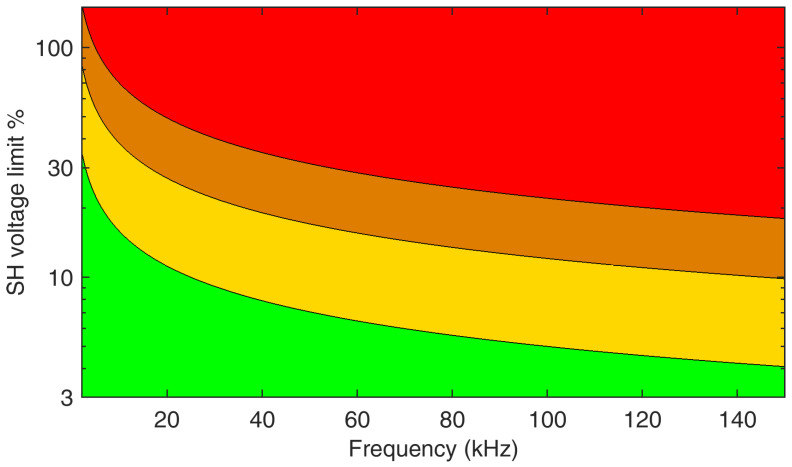
Limit curves for the percent SH voltage distortion based on the assumed $Q_{lim}=20$ of [20] and for *m* values covering m<=0.25, 0.25<m<=0.5, 0.5<m<=1.0, and m>1.0, with meaning of colors as in [20], namely “no risk”, “moderate risk”, “significant risk”, and “unacceptable”.

Limits can be derived for the stress on MV cable joints as outlined in [20] and sketched in Figure 6, having used the four curve profiles corresponding to “no risk” (m<=0.25), “moderate risk” (0.25<m<=0.5), “significant risk” (0.5<m<=1.0), and “unacceptable” (m>1.0). Provided that the reference value of 20 is correct and adequate to all situations, the derived limits (in terms of voltage distortion) seem to be less restrictive than those derived above for PLC interference and power losses.

#### 4.2.4. Interference with Energy Meters and Residual Current Devices

In this case, the tests available in the literature were not carried out with the purpose of defining a clear limit of distortion.

For energy meters, the extensive tests in [61] indicate that the rate of the rise of the input current affects the operation of the current sensing circuitry. The waveform in Figure 9 of [61] has a peak value of 50 A to 55 A with a stated rate of rise of 1.1 A/μs, leading to a rise time of about 20 μs; the duration is then in the order of 1 ms. The Fourier transform of such a waveform results in a spectrum component of about 0.8 A at 2 kHz (the beginning of the SH interval), multiplied by a grid impedance of some Ω, leads to some volts. This, however, does not mean that such an amount of distortion in the SH interval can lead to the observed malfunctions, as all spectral components must be in a precise phase relationship to reproduce the observed steep current impulse. The limitation of interference with energy meters thus becomes an issue of verification of compliance with an acceptable crest factor of the input current of CFL and LED devices by the respective manufacturers.

Desensitization was observed in some RCD models at about 800 mA, which, multiplied by some Ω of grid impedance, leads to some volts. The tested frequency values of the superposed disturbance were between 1 and 5 kHz, at which a higher frequency and even larger voltage distortion value are necessary for the same current to occur, giving a presumption of compatibility.

### 4.3. Assessment and Specifications for Instrument Transformers

SH distortion components relevant to interference and other negative effects were discussed in Section 2 and reference levels (both in terms of voltage and current at SH frequencies) were proposed earlier in this section. It is now worth verifying the current status of the measuring infrastructure in terms of the capability to quantify these phenomena, particularly at the MV level.

The aim of this section is to provide an overview of the current literature and standards concerning instrument transformers (ITs) in relation to supraharmonics. As a matter of fact, ITs are the elements of the grid meant to measure such power quality disturbances. However, since the SH issue became more relevant in recent years, ITs and their standards are not yet ready.

ITs play a crucial role in power systems, and their features are outlined in many standards like the well-known IEC 61869 series. In [85], general requirements are established, including provisions for Electromagnetic Compatibility (EMC) and specific tests like the Switching Impulse Voltage Test and EMC tests. For current transformers (CTs) detailed in [86], no additional requirements are specified; similarly, refs. [87,88,89] do not introduce extra conditions in the SH range for Inductive Voltage Transformers, Combined Transformers, and Low-Power Passive Voltage Transformers. However, IEC 61869-5 [90] highlights a relevant point regarding Capacitor Voltage Transformers, stating that they can be equipped with or without carrier-frequency accessories for power line carrier-frequency applications. IEC 61869-6 [91] introduces additional general requirements for the new generation of low-power instrument transformers (LPITs). It specifies tests for harmonic and inter-harmonic disturbance, conducted immunity tests at various frequency ranges, and radiated field immunity tests. These ensure the immunity of LPITs against a range of disturbances in the power supply. However, no additional information on SH testing is reported. The same comments can be extended to IEC 61869-9 [92], which focuses on the digital interface for instrument transformers, and IEC 61869-10 [93], which outlines frequency dependence and response characteristics, particularly relevant for Rogowski coils used in low-power passive current transformers. IEC 61869-14 [30] and IEC 61869-15 [94] specify accuracy tests on composite signals for current transformers and voltage transformers intended for DC applications, respectively. Overall, this short analysis clearly highlights the lack of consideration of SH phenomena inside the IEC 61869 series, confirming the need for new specific standards or a significant update of the existing ones.

**Figure 7:**
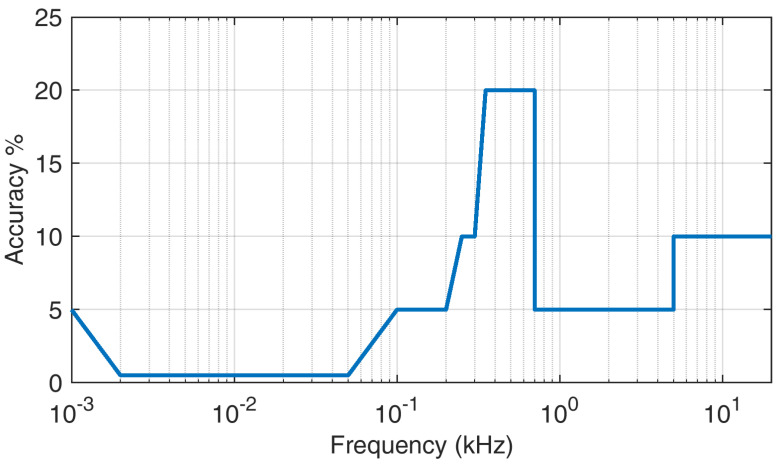
Accuracy limits vs. frequency as specified in the IEC 61869 standards (for the moment limited to 20 kHz).

Based on the above considerations, it is imperative to conduct a thorough evaluation of the measurement capabilities and related requirements for an IT in the SH frequency interval. These requirements can be categorized into three main aspects:

- *Frequency Response*: The frequency response of an IT poses a significant challenge when measuring supraharmonics. Most ITs and LPITs exhibit optimal performance only within a limited frequency interval. For example, inductive ITs are subject to resonances outside the traditional 50 Hz to 2500 Hz operating range; in addition, their response at higher frequencies may significantly deviate from the required flat profile, necessitating a comprehensive characterization process [95]. Although LPITs generally demonstrate better frequency performance, preliminary characterization remains indispensable.
- *Amplitude of the Measured Signal and Sensitivity*: Assessing the smaller SH amplitude proves challenging for ITs, which are inherently designed to achieve maximum accuracy at the rated voltage/current. Dealing with amplitudes that are three to six orders of magnitude lower than the nominal values presents a formidable task. This issue was already known during the evaluation of harmonic components up to the 50th harmonic. Some works in the literature investigated this way. For example, [96] addressed the topic, considering a very complex measurement chain consisting of sensors plus PMUs. Two potential solutions are conceivable: (a) installing an additional IT dedicated to measuring the SH frequency range with the discussed amplitudes, or (b) replacing all inadequate ITs with units capable of covering the entire frequency range between 50 Hz and 150 kHz, and whereas both solutions entail considerable challenges and expenses, their phased implementation over several years could align with the economic and physical constraints of the system operator.
- *Accuracy*: Measurement accuracy is not only another way of describing the sensitivity problem, it is also crucial when small and large signals combine onto the same IT sensor at the same time [97]. Non-linearity byproducts, as a consequence of mixing signal components of much different amplitude, can also limit the IT dynamic range, unless specific countermeasures are implemented [98] with the residual contributing to the overall accuracy. Figure 7 provides a summary of the current situation, derived from IEC 61869-6 [91] and related documents, for all frequency sub-intervals considered in the standard. The curve shows that accuracy limits presently extend up to 20 kHz, with an average accuracy ranging from 5% to 10%. Similar indications are extremely necessary for frequencies above 20 kHz to entirely cover the SH interval but need to be determined with a careful trade-off of all physical and practical limitations of these devices.

### 4.4. Conclusive Overview of Negative Effects and Proposed Limits

**Figure 8:**
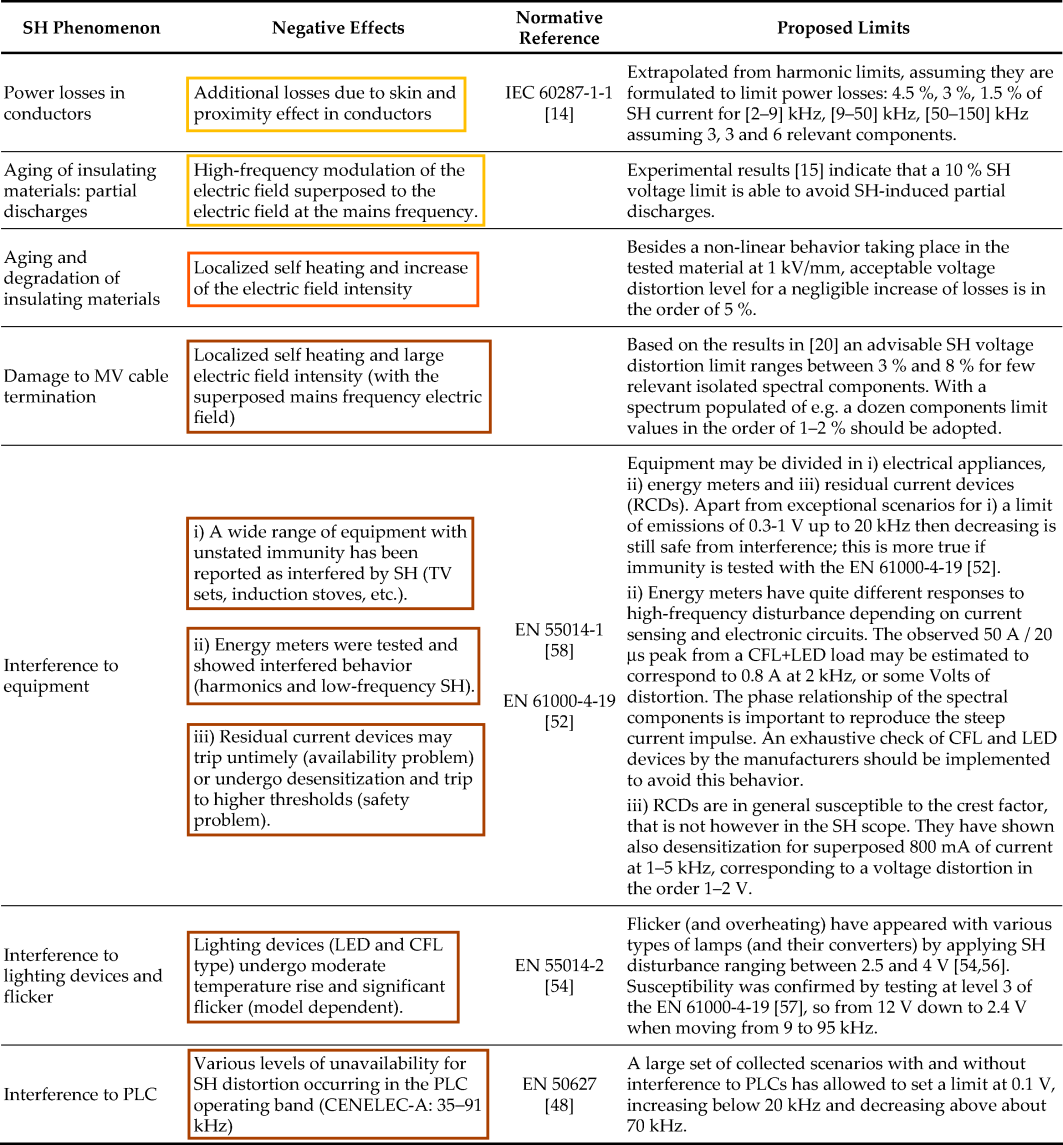
Overview of negative SH effects (in order of priority, or criticality, from **lowest** to **highest** and derived limits.

This section aims at concluding the discussion of documented negative effects and proposed limits summarizing the key points for the readers’ benefit. To avoid repetition and limit length, a large graphical table is used to provide an overview of the discussed phenomena (see Figure 8).

The identified limits are still not accurate and complete enough to take them as a direct input to standardization, but the objective has been achieved of evaluating the various negative effects for their significance and providing an indication of suitable distortion reference levels.

## 5. Conclusions

Supraharmonics are a new kind of distortion that is receiving more and more attention for a variety of reasons related to their intensity, time-frequency behavior, and sometimes unexpected negative effects. This work discussed the latter from different viewpoints: simple conduction losses (such as those caused by skin effect and proximity effect, verifying the validity of known formulas in the SH interval), dielectric losses and aging (including a particular form of stress to MV cable joints), influence on partial discharge phenomena (also causing aging of dielectrics), and interference with equipment, in particular to PLC devices. The objective was twofold: listing and describing all relevant negative effects in order to derive suitable limits for SH components and assessment methods, and, as a consequence, derive accuracy requirements and, in general, minimum performance requirements for measuring instruments (in particular ITs, considering the occurrence of many negative effects also at the MV level).

The size of the analyzed literature is significant and the overall set of works was reduced to 81 relevant items on which the present work is based. A few research items that are not strictly related to SH, but support the discussion, were not counted (such as the harmonic standards, works on power dissipation caused by harmonics, and so on).

Section 4 provided indications for limits with respect to interference with PLC, power losses, and MV cable termination stress, judged as the most relevant among the reviewed ones. The most conservative ones are the limits providing protection against interference, set to values between 0.1 V and 1 V, whereas power losses and MV cable termination stress are satisfactorily addressed with limits in the order of some % (corresponding to some Vs in LV grids and several tens of Vs, up to a hundred, for MV grids).

For a more accurate and reliable identification of suitable limits, a wider and more comprehensive set of data and well-documented interference cases is necessary, which will only be possible by continuing the experimental research on SH sources, in particular, renewables and EV charging stations. The experimental results provided in the literature have shown that time variability of emissions is a relevant and sometimes elusive characteristic of charging operations and renewable energy production. Apart from time variability, with respect to operating conditions, a second factor that increases the dimensionality of the problem is the dependency of emissions on grid impedance, also influenced by other connected loads and their operations. Since reproducibility, or the possibility of reuse, of the published measured data is extremely important, an effort is necessary to adopt wise choices of settings and parameters, as discussed in Section 4.1.

As introduced in Section 4.3, the measuring sensors (namely the instrument transformers) are becoming the limiting element of the uncertainty budget and they necessitate a research effort in two directions: better metrological characterization in conditions close to those of real use and improvement of the performance of devices in terms of the frequency range, sensitivity, and linearity. At the moment, ITs have standardized performance up to 20 kHz and accuracy specifications are such that in some cases, provided limits cannot be verified, not to mention assessing the contribution of single sources (at a fraction of the respective limits). The effort for IT standardization should thus be directed to extend the frequency interval to comprehensively cover SH phenomena and target the limits in the order of a fraction of V with less than 0.1% accuracy figures. Technically speaking, the latter may be achieved with new ITs by providing specific devices for the SH range (rejected by constructing the DC and mains fundamental component) or improved devices with dramatically improved dynamic range, accompanied, however, by digitizing systems with similar performance.

## Figures and Tables

**Table 1:** Overview of PLC operating bands.

Region	Standardization Body	Frequency Range
Europe	CENELEC	3 kHz to 95 kHz 95 kHz to 125 kHz 125 kHz to 140 kHz 140 kHz to 148.5 kHz
Japan	ARIB	10 kHz to 450 kHz
China	EPRI	3 kHz to 90 kHz 3 kHz to 500 kHz
USA	FCC	10 kHz to 490 kHz

**Table 2:** Reference harmonic limits for residential and industrial environments.

Frequency (Harm. Order)	Residential Harmonic Limits (A)	Industrial Harmonic Limits (A)
2	1.08	–
3	2.30	–
4	0.43	–
5	1.14	14
6	0.30	–
7	0.77	9
8	0.23	–
9	0.40	5
10	0.18	–
11	0.33	3
12	0.153	–
13	0.210	–
14	0.131	–
15	0.150	–
16	0.115	–
17	0.132	–
18	0.102	–
19	0.118	–
20	0.092	–
21	0.107	–
22	0.0836	–
23	0.0978	–
24	0.0767	–
25	0.0900	–
26	0.0708	–
27	0.0833	–
28	0.0657	–
29	0.0776	–
30	0.0613	–
31	0.0726	–
32	0.0575	–
33	0.0682	–
34	0.0541	–
35	0.0643	–
36	0.0511	–
37	0.0608	–
38	0.0484	–
39	0.0577	–
40	0.0460	–

## Data Availability

Data are contained within the article.
